# Evaluation of Riboflavin Intakes and Status of 20–64-Year-Old Adults in South Korea

**DOI:** 10.3390/nu7010253

**Published:** 2014-12-31

**Authors:** Ji Young Choi, Young-Nam Kim, Youn-OK Cho

**Affiliations:** Department of Food & Nutrition, Duksung Women’s University, Seoul 132-714, Korea; E-Mails: lovecjy730@hanmail.net (J.Y.C.); kyn3000@naver.com (Y.-N.K.)

**Keywords:** riboflavin intake, riboflavin status, urinary excretion, riboflavin supplements, Korean adults

## Abstract

A recent Korea National Health and Nutrition Survey indicated inadequate riboflavin intake in Koreans, but there is limited research regarding riboflavin status in South Korea. The purpose of this study was to determine riboflavin intake and status of Korean adults. Three consecutive 24-h food recalls were collected from 412 (145 men and 267 women) healthy adults, aged 20–64 years, living in South Korea and urine samples were collected from 149 subjects of all subjects. The dietary and total (dietary plus supplemental) riboflavin intake was 1.33 ± 0.34 and 2.87 ± 6.29 mg/day, respectively. Approximately 28% of the subjects consumed total riboflavin less than the Estimated Average Requirement. Urinary riboflavin excretion was 205.1 ± 190.1 μg/g creatinine. Total riboflavin intake was significantly positively correlated to the urinary riboflavin excretion. (*r* = 0.17171, *p* = 0.0363). About 11% of the Korean adults had urinary riboflavin <27 μg/g creatinine indicating a riboflavin deficiency and 21% had low status of riboflavin (27 μg/g creatinine ≤ urinary riboflavin < 80 μg/g creatinine). Thus, one-third of Korean adults in this study had inadequate riboflavin status. In some adults in Korea, consumption of riboflavin-rich food sources should be encouraged.

## 1. Introduction

Assessment of dietary intake is essential to investigate the relationships between diet and health in epidemiological studies and to design nutrient intervention studies. Particularly, determination of usual intake is critical to estimate the prevalence of inadequate intakes. The Korea Centers for Disease Control and Prevention have indicated that riboflavin is one of nutrients consumed inadequately by Koreans in the Korea National Health and Nutrition Survey (KNHANES) [1,2,3]. The KNHANES 2012 reported that a half of the Koreans consumed riboflavin less than the Estimated Average Requirement (EAR) for Koreans [3]. The KNHANES is a complex, stratified, multistage, probability-cluster survey of a representative sample of the non-institutionalized civilian Korean population. In the Nutrition Survey of KNHANES, one day of 24-h recall from participants is collected and dietary nutrient intakes calculated, which may not be captured as usual intake for some individuals with highly variable intakes. In addition, the results of nutrient intakes in KNHANES did not count the intake from dietary supplements, causing errors in the calculated nutrient intakes [4]. Thus, the prevalence of inadequate riboflavin intake in KNHANES might be overestimated.

Riboflavin—Vitamin B_2_ is a water-soluble vitamin that is involved for a number of oxidative enzyme systems in electron transport [4]. Riboflavin coenzymes, flavin mononucleotide and flavin adenine dinucleotide, are involved in diverse redox reactions to human metabolism as electron carriers [5], which play in the metabolism of energy, other B vitamins, drugs, and lipids [6,7]. A high prevalence of inadequate riboflavin intake and status has been reported in various population groups in many countries including USA, UK, France, Poland, and Japan [8,9,10,11]. Also, the recent studies have indicated that riboflavin deficiency increases risk of cancer at certain sites; lung, distal stomach, esophagus, and rectum [12,13,14,15,16]. Some epidemiological studies have identified relationships between cardiovascular diseases and diets low in riboflavin [17,18]. In South Korea, several studies reported dietary riboflavin intakes of Koreans [19,20], however, there is no study regarding current riboflavin status for Koreans including both intakes and biochemical measurements.

Therefore, the objectives of this study were to estimate total riboflavin intakes (dietary plus supplemental) and urinary riboflavin excretion as a biochemical measurement for riboflavin status and to evaluate current riboflavin status for Korean adults aged 20–64 years.

## 2. Experimental Section

### 2.1. Subjects

Four hundred and twelve healthy adults participated as subjects (145 men and 267 women), aged 20–64 years, living in the Seoul metropolitan area and the cities of Kwangju and Gumi, South Korea, during January 2010 to January 2012. Urine samples were collected from 149 adults (70 men and 79 women) who voluntarily provided the samples among 412 subjects. The subjects were recruited by advertisement in a convenience sampling of universities, gyms, and welfare centers. Adults who were not in good health, had known illnesses, or took medications were not included in the study. The Institutional Review Board of Duksung Women’s University approved the study (2010-1 & 2011-04-0001). Informed consent was obtained from each subject. The subjects were interviewed to obtain information regarding age, gender, current illness, medications taken, intake of vitamin and mineral supplements within 30 days of the interview, and appetite. All interviews were conducted by trained interviewers. Interviewers measured weights and heights of the subjects in light clothing and barefoot. Body mass index (BMI) was calculated as weight divided by squared height (kg/m^2^).

### 2.2. Calculation of Intakes of Selected Nutrients and Riboflavin

Three consecutive 24-h recalls (2 weekdays and 1 weekend day) were obtained from each subject by a trained interviewer. Intakes of macronutrients and vitamins were estimated using a computer-aided nutritional analysis program (CAN-Pro 4.0) developed by the Korean Nutrition Society [21]. The nutrient database of Can-pro 4.0 is based on the Korean Food Composition Table (Korean Rural Development Administration, 2006) and the Food Values (Korean Nutrition Society, 2009). The subjects consuming such supplements were asked to offer information on the names of the supplements and the amount, frequency, and duration of the use. Among 129 subjects taking any types of dietary supplements in this study, 80 subjects (19.4%, 31 men and 49 women) of all subjects took supplements containing riboflavin. In the subjects providing urine samples, 23% took riboflavin supplements. Thus, the amounts of riboflavin consumed by the subjects were reported as dietary riboflavin (from foods only) and total riboflavin intakes (dietary plus supplemental riboflavin). The EAR for riboflavin is 1.3 mg/day for men aged 19–64 years and 1.0 mg/day for women aged 19–64 years, respectively. The dietary and total intakes of riboflavin were compared with EARs for Koreans [22]. The top 10 major food sources of riboflavin consumed by the subjects were also determined.

### 2.3. Measurement of Urinary Riboflavin Excretion

First urine samples in the morning of an interview were collected from the 149 subjects in this study. Urine was protected from light and was kept in crushed ice. The samples were distributed in vials and can be stored at −70 °C until analysis. Urinary riboflavin was analyzed using the high-performance liquid chromatography (HPLC) method by Gatautis and Natio [23]. The standard of riboflavin was purchased from Sigma-Aldrich (St. Louis, MO, USA). All reagents were the HPLC grade in this study. The HPLC system consisted of UltiMate 3000 pump, UltiMate Injector and autosampler, FLD-3000 fluorescence detector (Thermo Scientific, Waltham, MA, USA), and C18 reversed-phase HPLC column (Waters, Ireland, 3.9 × 300 mm, 10 μm particle size). Riboflavin standard and urine were detected with λ_excitation_ of 450 nm and λ_emission_ 530 nm. Minimum detectable level was 0.0042 ng riboflavin. The coefficient of variance between assays was <5% for urine riboflavin. Urine creatinine was analyzed using an assay kit (Cayman Chemical Company, Ann Arbon, MI, USA). Interpretive criteria for the urinary excretion of riboflavin are <27 μg/g creatinine for deficient and 27–79 μg/g creatinine for low status [4].

### 2.4. Statistical Analysis

Data were analyzed by gender and by riboflavin supplementation using SAS version 9.1.3 software (SAS Institute, INC., Cary, NC, US). Values are reported as means ± standard deviations, and the differences by gender and by riboflavin supplementation (nonusers* vs.* users of riboflavin supplements) were analyzed using Student’s *t*-test. Histogram and Q-Q plots were used to determine whether the variables were normally distributed. Percentile values of riboflavin intakes were also reported by gender and by urinary riboflavin excretion. Pearson’s correlation coefficients were calculated to determine the correlations between riboflavin intake and urinary riboflavin excretion. Differences were considered significant *p* < 0.05.

## 3. Results

### 3.1. Subject Characteristics and Selected Nutrient Intakes

Table 1 shows general characteristics and selected nutrient intakes of 412 Korean adults aged 20–64 years. The mean age of all subjects was 38.8 ± 12.6 years and BMI was 22.9 ± 3.0 kg/m^2^. Energy intake of the subjects was 1866.9 ± 376.7 kcal/day. Energy intake of men was significantly higher than that of women (*p* < 0.001). Significantly higher mean intakes of macronutrients and selected vitamins were observed for men compared with women, except vitamin A and vitamin C (*p* < 0.001).

**Table 1:** General characteristics and selected nutrient intakes of 412 Korean adults by gender.

Variable	Men (*n* = 145)	Women (*n* = 267)	Total (*n* = 412)
Age (year) ^**^	36.6 ± 12.7	40.1 ± 12.4	38.8 ± 12.6
Weight (kg) ^***^	72.3 ± 9.8	56.8 ± 8.1	62.3 ± 11.4
Height (cm) ^***^	173.3 ± 6.0	159.9 ± 4.6	164.6 ± 8.2
BMI (kg/m^2^) ^***^	24.0 ± 2.7	22.2 ± 3.0	22.9 ± 3.0
*Macronutrients*			
Energy (kcal/day) ^***^	2119.5 ± 389.3	1729.7 ± 289.1	1866.9 ± 376.7
Carbohydrate (g/day) ^***^	275.3 ± 50.8	251.5 ± 54.0	259.9 ± 54.1
Protein (g/day) ^***^	89.7 ± 23.8	69.9 ± 15.7	76.9 ± 21.2
Total fat (g/day) ^***^	60.6 ± 18.6	48.7 ± 15.2	52.9 ± 17.5
*Vitamins*			
Vitamin A (μg RE ^1^)/day)	803.9 ± 302.1	813.7 ± 329.2	810.3 ± 319.6
Vitamin E (mg α-TE ^2^)/day) ^***^	19.4 ± 12.4	16.0 ± 5.1	17.2 ± 8.6
Thiamin (mg/day) ^***^	1.4 ± 0.4	1.2 ± 0.3	1.2 ± 0.3
Niacin (mg NE ^3^)/day) ^***^	19.8 ± 5.5	15.9 ± 4.0	17.3 ± 4.9
Vitamin B_6_ (mg/day) ^***^	1.9 ± 0.6	1.8 ± 0.6	1.8 ± 0.5
Vitamin C (mg/day) ^***^	96.5 ± 45.8	119.9 ± 63.0	111.6 ± 58.5

Values are means ± standard deviations; ^**^ *p* < 0.01, ^***^ *p* < 0.001 by *t*-test; ^1^ Retinol Equivalent; ^2^ α-Tocopherol Equivalent; ^3^ Niacin Equivalent.

### 3.2. Riboflavin Intakes

The dietary and total riboflavin intake of the subjects was 1.33 ± 0.34 mg/day and 2.87 ± 6.29 mg/day, respectively (Table 2) and there were no significant differences in the intakes by gender (*p* ≥ 0.05). The ratio of dietary riboflavin intake to energy intake of women was significantly higher than that of men (*p* < 0.001). Users of riboflavin supplements consumed more total riboflavin than nonusers (*p* < 0.001). Approximately 42.8% of men and 29.2% of women had dietary riboflavin intakes less than EAR, but the prevalence of inadequate riboflavin intake was reduced by counting riboflavin supplements to 33.8% in men and 24.7% in women. Only two subjects taking riboflavin supplements had total riboflavin intakes less than EAR. The top 10 major dietary sources of riboflavin consumed by the subjects were whole egg, citrus fruit, whole milk, Ra Myeon (Korean instant noodle), Kimchi, pork (loin), mackerel, spinach, chicken, and pork (belly). There were no riboflavin fortified foods that the subjects consumed except cereals. Cereal was the 27th largest source of riboflavin in this study and approximately 2.7% of the subjects consumed cereals.

### 3.3. Urinary Riboflavin Excretion and Riboflavin Status

Urinary riboflavin excretion was 205.1 ± 190.1 μg/g creatinine (Table 3). No significant difference in riboflavin excretion was observed by gender (*p* ≥ 0.05), but the excretion of users of riboflavin supplements was significantly higher than that of nonusers (*p* < 0.001). Approximately 11% of the Korean adults had a riboflavin concentration <27 μg/g creatinine indicating a biochemical deficiency of riboflavin. The subjects having low status (27 μg/g creatinine ≤ urinary riboflavin < 80 μg/g creatinine) were 20.8% of all subjects and 8.8% of the users.

### 3.4. Percentile Values of Riboflavin Intake

Percentile values of dietary and total riboflavin intakes by gender and by urinary riboflavin excretion are shown in Table 4. Median dietary and total riboflavin intakes of men were 1.37 and 1.46 mg/day, respectively. Women had median dietary and total riboflavin intakes of 1.18 and 1.24 mg/day, respectively. Median dietary and total riboflavin intakes of men with urinary riboflavin ≥27 μg/g creatinine were 1.37 and 1.44 mg/day, respectively. Median dietary and total riboflavin intakes of women with urinary riboflavin ≥27 μg/g creatinine were 1.33 and 1.43 mg/day, respectively.

### 3.5. Associations among Riboflavin Intakes and Urinary Riboflavin Excretion

There was no significant correlation between urinary riboflavin and dietary riboflavin intakes including the ratio of dietary riboflavin to energy intake (*p* ≥ 0.05) (Table 5). However, urinary riboflavin excretion was significantly positively correlated with total riboflavin intake (*r* = 0.17171, *p* = 0.0363).

## 4. Discussion

Recent studies have reported the relationships between riboflavin status and some diseases such as certain types of cancers [12,13,14,15,16] and cardiovascular disease [18]. Riboflavin is involved in the folate-mediated one-carbon. Thus, poor riboflavin status may lead to an elevated rate of DNA damage and altered methylation of DNA, both of which are important risk factors for cancer [24]. Inadequate riboflavin intake has been indicated by a national dietary survey of several countries [25,26,27] including South Korea [1,2,3]. However, in Korea, a study regarding riboflavin status for Korean adults is very limited. Therefore, this study estimated intakes and urinary excretion of riboflavin and evaluated riboflavin status of 20–64-year-old adults living in the Seoul metropolitan area and the cities of Kwangju and Gumi, South Korea.

**Table 2:** Riboflavin intakes of 412 Korean adults by gender and by riboflavin supplementation.

Variable	Gender		Riboflavin Supplementation		Total (*n* = 412)
	Men (*n* = 145)	Women (*n* = 267)	Nonusers of Riboflavin Supplements (*n* = 332)	Users of Riboflavin Supplements (*n* = 80)	
Dietary riboflavin intake (mg/day)	1.35 ± 0.34	1.31 ± 0.33	1.25 ± 0.35	1.27 ± 0.32	1.33 ± 0.34
Dietary riboflavin/energy (mg/1000 kcal)	0.65 ± 0.14 ^***^	0.74 ± 0.16	0.68 ± 0.16	0.67 ± 0.15	0.69 ± 0.16
Total riboflavin intake (diet + supplements) (mg/day)	2.62 ± 5.51	3.08 ± 6.94	1.25 ± 0.35 ^***^	5.72 ± 9.31	2.87 ± 6.29
Using supplements with riboflavin (% (*n*))	21.4 (31)	18.4 (49)	0 (0)	100 (80)	19.4 (80)
Not meeting the Estimated Average Requirement with dietary riboflavin (% (*n*))	42.8 (62)	29.2 (78)	33.7 (112)	35.0 (28)	33.9 (140)
Not meeting the Estimated Average Requirement with total riboflavin (% (*n*))	33.8 (49)	24.7 (66)	33.7 (112)	2.5 (2)	27.6 (114)

Values are means ± standard deviations; The Estimated Average Requirement for riboflavin is 1.3 mg/day for men aged 19–64 years and 1.0 mg/day for women aged 19–64 years; ^***^ *p* < 0.001 by *t*-test.

**Table 3:** Urinary riboflavin excretion of 149 Korean adults by gender and by riboflavin supplementation.

Variable	Gender		Riboflavin Supplementation		Total (*n* = 149)
	Men (*n* = 70)	Women (*n* = 79)	Nonusers of RiboflavinSupplements (*n* = 115)	Users of RiboflavinSupplements (*n* = 34)	
Urinary riboflavin (μg/g creatinine)	193.8 ± 183.3	215.1 ± 196.4	175.8 ± 164.2 ^***^	304.3 ± 236.2	205.1 ± 190.1
27 μg/g creatinine ≤ Urinary riboflavin < 80 μg/g creatinine (%(*n*))	21.4 (15)	20.3 (16)	24.3 (28)	8.8 (3)	20.8 (31)
Urinary riboflavin < 27 μg/g creatinine (%(*n*))	12.9 (9)	10.1 (8)	11.3 (13)	11.8 (4)	11.4 (17)

Values are means ± standard deviations. ^***^ *p* < 0.001 by *t*-test.

**Table 4:** Percentile values of dietary and total riboflavin intakes of Korean adults.

Subject	*n*	Dietary Riboflavin (mg/day)					Total Riboflavin ^1^ (mg/day)				
		5th	25th	50th	75th	95th	5th	25th	50th	75th	95th
*Total subjects (n* *= 412)*											
Men	145	0.76	1.11	1.37	1.58	1.96	0.77	1.16	1.46	1.83	3.76
Women	267	0.71	0.95	1.18	1.39	1.78	0.72	1.00	1.24	1.63	2.94
*Selected subjects (n=149)* ^2^											
Total men	70	0.73	1.08	1.38	1.60	1.96	0.76	1.29	1.47	1.77	6.33
Men with urinary riboflavin ≥ 27 μg/g creatinine	61	0.73	1.08	1.37	1.54	1.93	0.76	1.19	1.44	1.66	6.33
Total women	79	0.71	1.09	1.30	1.51	1.97	0.81	1.11	1.39	1.78	21.31
Women with urinary riboflavin ≥ 27 μg/g creatinine	71	0.81	1.11	1.33	1.53	1.97	0.93	1.17	1.43	1.78	5.20

^1^ Dietary + supplemental riboflavin; ^2^ Subjects providing urine sample for urine riboflavin analysis.

**Table 5:** Correlations between riboflavin intakes and urinary riboflavin excretion.

Riboflavin Intake	Urinary Riboflavin
Dietary riboflavin	0.082 (0.318) ^1^
Dietary riboflavin per energy	0.129 (0.116)
Total riboflavin (diet + supplements)	0.171 (0.036) ^*^

^1^ *p*-value; ^*^ significant at *p* < 0.05.

In this study, the mean dietary riboflavin intake (1.33 mg/day) is similar to recently reported dietary riboflavin intake of 20–64-year-old adults reported in KNHANES 2010–2012 [3]. However, dietary riboflavin intakes of Koreans were lower than those of Chinese adults (1.6 mg/day) [25], American adults (2.19 mg/day) [28], and British adults (1.97 mg/day for men and 1.50 mg/day for women) [26]. In the current study, dietary supplements providing riboflavin increased mean intake from food sources alone by 21% for men, from 1.35–2.62 mg, and by 25% for women, from 1.31–3.08 mg. Approximately 19% of the subjects took riboflavin supplements and their mean total riboflavin intake was 5.72 mg/day, much lower than that of American adults taking riboflavin supplements (11.23–11.61 mg/day) [28]. In this study, a 24-h recall method was used for calculation of riboflavin intake. However, food frequency questionnaire or food diary methods were used for estimation of riboflavin intake in other countries [26,27,28].

The Korean Dietary Reference Intakes (KDRIs) for riboflavin are set as EAR and Recommended Nutrient Intake in all age groups older than one year old. EAR is the daily nutrient intake estimated to meet the requirement of half of healthy individuals in a life-stage group, thus are set at the median of the distribution or requirements. EAR is used to estimate the prevalence of inadequate intake within a group of individuals. EAR of e KDRIs for riboflavin is set at 1.3 mg/day for men and 1.0 mg/day for women aged 19–64 years, based on reports that the riboflavin requirement is at least more than 1 mg/day in Koreans to maintain normal urinary riboflavin excretion and based on median intake of Korean adults in KNHANES 2007 [22]. In this study, median (50th percentile) dietary riboflavin intake of men and women was 1.37 and 1.18 mg/day, respectively. Median dietary intake of men and women with urinary riboflavin ≥27 μg/g creatinine, a cut-off point of riboflavin deficiency, was 1.37 and 1.33 mg/day, respectively. Thus, current riboflavin EAR for men of KDRIs may be appropriate, but EAR for women might be underestimated. KNHANES 2010–2012 [3] reported that about 45% of men (*n* = 3127) and 46% of women (*n* = 4081) consumed the dietary riboflavin less than EAR. In the current study, a low proportion of participants (33.9%) had dietary riboflavin intakes below EAR and the proportion for total (dietary + supplemental) riboflavin intake was much lowered (27.6%). In the US, the prevalence of American adults consuming dietary riboflavin less than Recommended Dietary Allowance was 3% [28]. In British adults, 8% had total riboflavin intake less than the Reference Nutrient Intakes, a nutrient requirement to meet the needs of 97%–98% of healthy individuals [22,26]. Therefore, the prevalence of inadequate riboflavin intake with riboflavin supplementation in this study was much lower than that of KNHANES. However, the prevalence of inadequate intake in Korean adults was still high compared to the prevalence of other countries.

Balance studies in human subjects show clearly that as riboflavin intake increases there is a progressive rise in urinary excretion of riboflavin [29]. Urinary excretion of riboflavin reflects an excess of current intake over tissue requirement and the measurement of urinary excretion provides useful information regarding tissue saturation [18,29,30] under the circumstance of optimum nutritional status for riboflavin. Thus, urinary riboflavin excretion could be used as a potential biomarker to assess its mean estimated intakes in a group. [30,31,32,33]. Urinary losses decrease when riboflavin stores decrease. Besides the urinary excretion, the determination of erythrocyte glutathione reductase activity coefficient (EGRAC) is also used. EGRAC is sensitive to changes in riboflavin intake up to levels of intake approaching tissue saturation of riboflavin. However, differences in the relationship between intake and EGRAC values among different population groups have indicated [33] that re-evaluation of EGRAC threshold for riboflavin deficiency should be considered [34]. In this study, urinary excretion was used for evaluation of riboflavin status. The study conducted by Lim and Yoon [20] in 1997 reported that 8.8% and 14.2% of Korean women (*n* = 38) had riboflavin deficient and low status, respectively, based on urinary excretion, which is in line with the prevalence of riboflavin deficiency and low status in Korean adults of this study. The national survey of UK in 2014 indicated the rate of riboflavin deficiency was 69% of British adults [26]. Whitfield *et al.* [35] reported that 67% of Canadian women (*n* = 51) had a suboptimal (low) status in 2014. The difference in rates of inadequate riboflavin status between the current study and the studies of UK and Canada may be due to a use of different biochemical index, EGRAC, in UK and Canadian studies. In this study, seven subjects (20%) of users of riboflavin supplements had inadequate riboflavin status. The mean riboflavin intake only from supplements of users with normal status was 6.80 mg/day (0.36–40 mg/day), but those of users with deficiency and low status was 0.94 mg/day (0.51–1.6 mg/day). Additional riboflavin intakes from supplements were low in users with inadequate status; therefore, it seems that the intake from supplements did not affect riboflavin status of them.

A positive relationship has been observed between riboflavin intake and urinary excretion [31,36]. In this study, no significant correlation was observed between dietary riboflavin intake and urinary riboflavin. However, urinary riboflavin excretion showed a positive correlation with total riboflavin intake (*r* = 0.1717, *p* < 0.0363). Because the subjects supplementing riboflavin additionally consumed 0.36–40 mg/day of riboflavin, total riboflavin intake showed significant correlation with urinary riboflavin rather than dietary intake. These findings show that the urinary riboflavin level reflects riboflavin intake and the intake in population affects their riboflavin status as well. The nutrient database in this study is based on raw foods. In raw foods, nutrient contents are changed by food preparation, cooking conditions (e.g., time and temperature), and the addition of different ingredients depending on household preferences. Therefore, riboflavin cooked foods consumed by the subjects might be underestimated [37]. Three consecutive 24-h recalls were obtained from each subject, which may not be captured as usual intake. Thus, the correlation between urinary riboflavin excretion and total riboflavin intake might not be strong.

## 5. Conclusions

In Korean adults of this study, the prevalence of inadequate riboflavin intake including riboflavin supplementation was much lower than that of KNHANES of South Korea, but the prevalence of inadequacy in Korean adults was still high compared to other countries. One-third of Korean adults in the current study had inadequate riboflavin status, thus consumption of good sources of riboflavin should be encouraged in Koreans such as milk and milk products, whole egg, pork, and mandarins.
